# Divergent Impact of Breast Cancer Laterality on Clinicopathological, Angiogenic, and Hemostatic Profiles: A Potential Role of Tumor Localization in Future Outcomes

**DOI:** 10.3390/jcm9061708

**Published:** 2020-06-02

**Authors:** Ruszkowska-Ciastek Barbara, Rhone Piotr, Bielawski Kornel, Zarychta Elżbieta, Rość Danuta, Nava Eduardo

**Affiliations:** 1 Department of Pathophysiology, Faculty of Pharmacy, Nicolaus Copernicus University, Collegium Medicum in Bydgoszcz, 85-796 Bydgoszcz, Poland; kornel-bielawski@wp.pl (B.K.); erhone@gmail.com (Z.E.); drosc@cm.umk.pl (R.D.); 2 Clinical Ward of Breast Cancer and Reconstructive Surgery, Oncology Centre Prof. F. Łukaszczyk Memorial Hospital, 85-796 Bydgoszcz, Poland; prhone@wp.pl; 3 Second Department of Obstetrics and Gynecology, Centre of Postgraduate Medical Education, 01-813 Warsaw, Poland; 4 Area of Physiology, Department of Medical Sciences, University of Castilla-La Mancha, School of Medicine and Regional Centre for Biomedical Research (CRIB), E-02006 Albacete, Spain; Eduardo.Nava@uclm.es

**Keywords:** breast cancer laterality, angiogenesis, hemostasis, molecular determinants of breast cancer, metastasis

## Abstract

To date, lateral differences of invasive breast cancer (IBrC) with respect to the angiogenic and hemostatic profiles were never studied. Here, we aimed to determine the relationship of tumor laterality with various clinical and pathological parameters including angiogenic and hemostatic profiles. A total of 92 women that were initially non-metastatic and treated by surgery were included in this single-center prospective study. Patients were grouped according to tumor localization. A four-year follow-up was accomplished in all patients with a 15.22% recurrence rate. An immunoassay of selected angiogenic and hemostatic parameters, as well as immunohistochemistry of estrogen and progesterone receptors, human epidermal growth factor receptor 2 (HER2), and Ki67, was comparatively performed in groups with right- and left-sided IBrC. The same analysis was carried out in a subgroup of patients with luminal A molecular subtype of cancer. Patients with right-sided tumors free of nodal involvement had a significantly longer overall survival compared to their left-sided counterparts (*p* = 0.0491). Additionally, right-sided tumors had a higher predisposition to be a luminal-A subtype of IBrC (*p* = 0.0016). Furthermore, 10% of left-sided tumors exhibited an overexpression of HER2, while only 2% patients suffering right-sided tumors displayed a positive score (*p* = 0.0357). Our findings revealed a significantly higher concentration of vascular endothelial growth factor (VEGF)-A (*p* = 0.0136), lower anti-angiogenic ratios (sVEGFR1/VEGF-A (*p* = 0.0208) and sVEGFR2/VEGF-A (*p* = 0.0068)), and elevated plasminogen activator inhibitor type 1 (PAI-1) (*p* = 0.0229) in patients with breast cancer localized in the left breast, regardless of the molecular subtype of IBrC. Our study showed that left-sided breast tumors without lymph node metastases demonstrate worse overall survival. Laterality of IBrC is associated with pro-angiogenic and pro-thrombotic conditions. We propose to consider laterality as a prognostic factor of IBrC.

## 1. Introduction

According to literature data, breast neoplasms ordinarily develop unilaterally [1,2]. Dysregulation of developmental stability or fluctuating asymmetry between left and right breasts may lead to cancer creation in one breast [2]. Laterality is defined as left–right asymmetry in paired organs. Practically nothing is known about the etiopathogenesis of cancer laterality [3]. The existence of this phenomenon was proposed by von Fellenberg in the first half of the 20th century, who observed a greater frequency of breast cancer in the left breast [1]. Since then, it remains a questionable issue. The question arises as to why a tumor develops just in one breast when both mammary glands are equally exposed to risk factors [2]. 

Right-sided cancers are more likely to be diagnosed in lungs, testes, ovaries, and kidneys, while breast cancer and melanoma demonstrate left-sided location dominance, i.e., melanoma exhibits 10% higher prevalence on the left side of the body. However, the increased occurrence of left-sided tumors has no impact on overall survival [3,4,5]. In contrast to those findings, Erendeeva et al. observed that the survival of patients with left-sided invasive breast cancer (IBrC) was higher than subjects with right-sided ones [6]. Various theories were proposed to explain this phenomenon [3,4,5]. The first of these is based on the fact that the left breast is slightly larger than the right one. However, this hypothesis was rejected due to the fact that the breast size depends on the body mass index, the age, and parity status. The next theory assumes that right-handed women check their left breasts more accurately. Other hypotheses linked Erendeeva’s observation to breastfeeding patterns or even sleeping habits having an impact on this issue [3,4,5]. Furthermore, mammary tissue is very sensitive to hormonal variability and shows changes during follicular and luteal phases of the menstrual cycle, modifying periodic breast density [2,7]. In this line, certain women are more sensitive to “fluctuating asymmetry” and, consequently, one breast is more susceptible to cancer development [2]. 

The conventional clinical approach to breast cancer diagnosis, treatment patterns, and prognosis takes into account age, menopausal status, tumor (T)/node (N)/metastasis (M) classification, histological grade, tumor size, axillary lymph node status, Ki67 expression, hormone receptors, and human epidermal growth factor receptor 2 (HER2) status [8]. However, tumor location does not play a fundamental role in this regard. Rather, tumor location is considered relevant for the possible consequences of radiotherapy and subsequent cardiovascular disease (including myocardial infarction and stroke) [4]. Despite the fact that current radiotherapy is much safer than it was formerly, the left side of the body is inevitably more exposed to the negative effects of radiation.

Interactions between angiogenesis-regulating factors and hemostatic mediators are necessary for the malignant transformation and nutrition of tumors. Protease-activated receptor (PAR) signaling is importantly involved in this interaction. The metastatic behavior of breast cancer cells is promoted by PAR-2 stimulation. Tissue factor (TF), a well-known stimulator of tumor angiogenesis, invasion, and metastasis [9,10], upregulates vascular endothelial growth factor (VEGF) and, therefore, vascular neoformation [11]. Indeed, VEGF is involved in malignant and non-malignant angiogenesis [10]. VEGF transmits its pro-angiogenic signals by means of tyrosine kinase receptors, VEGFR1 (Flt-1), VEGFR2 (Flk-1), and VEGFR3 (Flt-4). On the other hand, soluble VEGF receptor forms, sVEGFR1 (sFlt-1) and sVEGFR2 (sFlk-1), predominantly act as suppressors of VEGF’s pro-angiogenic effects [10,11]. 

To our knowledge, lateral differences in breast cancer were never studied at a molecular level. The present work analyzes angiogenic and hemostatic profiles in right- and left-sided invasive breast cancer subjects. Our aims were (1) to unveil a possible lateralization with regard to HER2 expression, lymph node involvement, or molecular subtypes of breast cancer, (2) to examine left–right asymmetries with respect to angiogenic, hemostatic, and inflammatory mediators in patients with primary, invasive breast cancer, and (3) to explore whether the tumor laterality can predict disease recurrence. 

## 2. Material and Methods

### 2.1. Ethical Approval and Consent

The study was approved by the University ethical committee (permission no. KB 547/2015). All subjects provided written informed consent to participate in this study after a full explanation of the study. All procedures were designed, conducted, and reported in compliance with the Declaration of Helsinki. Data obtained during the course of this study were accessible to the investigators only. All information was coded by number, and no name was noted.

### 2.2. Population Sampling

A total of 92 patients treated surgically for unilateral, primary, invasive breast carcinoma (IBrC) from the Clinical Ward of Breast Cancer and Reconstructive Surgery, Oncology Center, Prof. F. Łukaszczyk Memorial Hospital, Bydgoszcz, Poland were included into this prospective cohort study. The median age at the day of diagnosis was 54.5 years (interquartile range—IQR: 44–66). All the subjects were asked to complete a questionnaire including date of birth, parity, date of first and last menarche, weight, height, smoking status, alcohol intake history, past medical history including any previous history of thromboembolic events, other disorders, and any drug intake. Smoking status was categorized as “smoker” and “currently non-smoker”. Body mass index (BMI) was calculated as self-reported weight (kg) divided by the square of self-reported height (in meters). According to the World Health Organization definition, patients were classified into three BMI subgroups: normal weight (18.5 to 24.9 kg/m^2^), overweight (25.0 to 29.9 kg/m^2^), obese (>30.0 kg/m^2^). Full details of the study recruitment and procedures were previously reported [12].

### 2.3. Exclusion Criteria for Invasive Breast Cancer Cohort

Patients with at least one of the following characteristics were excluded: (a) male gender, (b) age below 40 and over 70 years, (c) carcinoma in situ, (d) neoadjuvant therapy, (e) bilateral IBrC, (f) tumor size >5 cm, (g) stage IIIA or higher, (h) previous diagnosis of any cancer type, (i) distant metastasis, (j) incomplete histopathology details of primary tumor, (k) overt diabetes mellitus (DM) or impaired glucose tolerance, (l) recent bleeding or thrombotic events, and (m) chronic inflammatory diseases or autoimmune disease. 

### 2.4. Clinical and Pathological Reports

Primary, non-metastatic tumor laterality was classified as left-sided and right-sided. Right-sided breast cancer was diagnosed in 45 cases, while 47 cases were diagnosed in the left breast. Clinicopathological data including histology type, histological grade, tumor size, nodal status, estrogen and progesterone receptors, and human epidermal growth factor receptor 2 (HER2) status were collected and compared between patients based on tumor laterality (Table 1). Patients were stratified by molecular subtype according to immunohistochemical marker profiles. Details of adjuvant therapy were also compared between groups. These included the administration of adjuvant chemotherapy, immunotherapy, hormonal therapy, or a combination, as well as radiation therapy details including primary target (breast vs. breast and regional nodes, use of boost, and prescribed dose). None of the patients were taking any drugs with hemostatic and fibrinolytic effects [12]. 

### 2.5. Follow-Up Details 

The median follow-up time after the index date was 42 months (IQR: 36–44) with a 15.22% recurrence rate. For the progression-free survival analysis, 14 events were noted, including three (3.26%) loco-regional recurrences, three (3.26%) distant metastases, and eight (8.69%) deaths. The follow-up of these patients consisted of clinical evaluation (breast and lymph node palpation), laboratory assessments (blood biochemical), breast ultrasonography, liver ultrasound, mammography, and other suitable examinations. Relapse was established as signs of metastatic disease or local recurrence as confirmed by positron-emission tomography (PET)/computed tomography (CT) or death (excluding deaths unrelated to the disease).

### 2.6. Measurement of Analyzed Parameters

Blood collection was intravenously performed in the morning after overnight fasting (12 h) according to clinical standards. Blood was collected using ethylenediaminetetraacetic acid (EDTA)-coated anticoagulant BD Vacutainer^®^ tubes and 4.5-mL tubes (BD Vacutainer^®^, Belliver Industrial Estate, Plymouth, UK) containing 0.105 M buffered trisodium citrate following the manufacturer’s instructions. Citrate and EDTA plasma aliquots were centrifuged and then stored at −80 °C until analysis. Plasma activities or concentrations of tissue factor (TF), tissue factor pathway inhibitor (TFPI), tissue plasminogen activator (t-PA), and plasminogen activator inhibitor type 1 (PAI-1) were measured using the standard immunoassay technique in accordance with the guidelines of the manufacturer. Specific details of parameter assessments were previously reported [13]. EDTA-plasma concentrations of VEGF-A, soluble form of VEGF receptors type 1 and 2 (VEGF, sVEGFR1/Flt-1, sVEGFR2/KDR Immunoassay Test, Quantikine, R&D systems, USA), heparanase, stromal cell-derived factor 1 (SDF-α) (ELISA Kit for Heparanase (HPA), SDF-α, Cloud-Colne Corp., TX, USA), YKL-40 protein (Human Chitinase 3-like 1 ELISA, BioVendor Research and Diagnostic products, Brno-Řečkovice a Mokrá Hora, Czech Republic), and von Willebrand Factor (Imubind^®^vWF ELISA, BioMedica Diagnostics, WA, USA) were measured in all samples using the enzyme-linked immunosorbent assay (ELISA) kits, where the reaction mixture was added to a 96-well plate according to the manufacturer’s specifications. Assays were run by personnel with no access to the clinical data of the patients.

### 2.7. Immunohistochemical (IHC) Analyses

Tumor blocks for each case were studied using immunohistochemical staining for estrogen and progesterone receptors (ER and PR, respectively), Ki67 expression, and human epidermal growth factor receptor 2 (HER2) scoring using commercial methods. A 2+ score was considered as equivocal and was tested for HER2 gene amplification by fluorescent in situ hybridization techniques (FISH), in accordance with the manufacturer’s instructions. Hormone receptor status was classified as a two-level variable, namely, estrogen receptor-positive and/or progesterone receptor-positive, as well as both estrogen receptor-negative and progesterone receptor-negative. Detailed procedures are included in our previous work [13].

### 2.8. Statistical Analysis

Demographics including age, clinicopathological determinants, and treatment procedures were compared between patients with left-sided versus right-sided cancers by use of Pearson’s χ^2^ test. The χ^2^ test was used to examine the relationship between qualitative variables. Normality of distribution was assessed using the Shapiro–Wilk test. The differences between angiogenic, hemostatic, and other parameters were evaluated using the Mann–Whitney U test. Continuous variables were presented as median and interquartile range (IQR) values where appropriate. Categorical variables were expressed as counts or percentages. The survival curves were estimated with the Kaplan–Meyer product limit method. Progression-free survival (PFS) was calculated from the time of initial surgery to the first radiological evidence of recurrence. Overall survival was calculated from the start of palliative treatment to the date of death. Cox’s proportional hazards models were used to analyze the risk of disease recurrence at any time over four years of follow-up. Results were summarized as hazard ratios and 95% confidence intervals. Prognostic values of angiogenic and hemostatic parameters with respect to disease relapse were calculated by linear regression. Statistical analysis was performed with the software of Statistica, version 12. (StatSoft, Cracow, Poland). A probability (*p*) < 0.05 was considered to be statistically significant. 

## 3. Results

### 3.1. Clinical Presentation of Patients with Regard to Breast Cancer Laterality

Table 1 (T1) summarizes all the clinical and anthropometric parameters of the study with respect to tumor localization. A total of 92 subjects were enrolled into the trial (T1.1). All patients had primary, unilateral, non-metastatic invasive breast cancer. All patients were of Slavic descent. All the cases occurred between the fourth and seventh decades of life (IQR: 44–66), with the median patient age at the time of the initial diagnosis of cancer being 54.5 years (T1.2). Clinical, tumor, and treatment characteristics were compared between laterality groups. The prevalence of left versus right-sided tumors had the same proportion among the IBrC patients. 

#### 3.1.1. Tumor Laterality According to Age, Body Mass Index, Smoking, Menopausal Status, and Clinical and Histological Classifications 

The laterality ratio (left/right ratio, LRR) was measured as the number of primary breast tumors diagnosed in the left breast divided by those recognized in the right breast (1.04). Thus, the prevalence of left- and right-sided IBrC was similar. Menopausal status (T1.3) was determined in all patients and showed 30 (33%) pre-menopausal and 62 (67%) post-menopausal cases. There were no significant differences between left-sided versus right-sided breast cancers with regard to age, menopausal status, body mass index (BMI), smoking habits, or clinical and histological classifications of the tumor (T1.2–7). The vast majority of invasive cancers were duct cell (82; 89%), followed by lobular (10; 11%) (T1.7).

#### 3.1.2. Tumor Laterality According to Histological Grading System

Grading estimated by means of the Elston–Ellis system (T1.8) showed that most tumors were moderately differentiated, i.e., G2 (67; 73%). These were followed by low-differentiated, i.e., G3 (19; 21%), and well-differentiated tumors, i.e., G1 (6; 6%) (*p* = 0.0698). Patients with G2 of IBrC had the tumor localized in the right breast, with a left-to-right (LRR) ratio of 0.81. In contrast, tumors with lower or higher score were observed in left-sided IBrC (LRR: 5 and 1.71, respectively). 

#### 3.1.3. HER2 Expression According to Tumor Laterality

Breast cancer laterality was also associated with HER2 expression (*p* = 0.0357). HER2 status (T1.9) was negative in 81 patients (88%). While nine (10%) left-sided tumors exhibited an overexpression of HER2, only two (2%) patients with right-sided tumors displayed a positive score (LRR = 4.5). 

#### 3.1.4. Molecular Subtype of Breast Cancer According to Tumor Laterality

Regarding tumor molecular subtypes (T1.10), IBrC laterality showed a significant association (*p* = 0.0016). Right-sided tumors had a higher predisposition to be luminal-A (ER^+^ PR^+^ HER2^−^, Ki67 < 20%) subtypes of IBrC (40% vs. 26% of the left sided; LRR = 0.65). In contrast, left-sided tumors were positive for other molecular subtypes including luminal-B HER2 negative (ER^+^ PR^+/−^ HER2^−^, Ki67 > 20%), luminal-B HER2 positive (ER^+^ PR^+/−^ HER2^+^, Ki67 all values], non-luminal HER2 positive (ER^−^ PR^−^ HER2^+^, Ki67 all values), or basal-like subtype (BLBC) (ER^−^ PR^−^ HER2^−^, Ki67 all values) (23 vs. 8 cases, respectively; LRR = 2.87). 

#### 3.1.5. Lymph Node Status According to Tumor Laterality

With respect to the axillary lymph node status (T1.11), among the 72 node-free patients, 42 (58%) had left-breast cancer and 30 (42%) had right-breast cancer. Among the 20 cases with confirmed nodal metastases, 15 (75%) had right-sided breast cancer and five (25%) had left-sided IBrC (LRR = 0.33 (*p* = 0.0083)). 

### 3.2. Treatment Profile of Patients

Details of the type of surgery procedures and adjuvant therapy were also compared between groups. These included the administration of adjuvant chemotherapy, immunotherapy (trastuzumab, a humanized anti-HER2 monoclonal antibody), radiation, and hormonal therapy in all IBrC subjects. From a total of 92 patients, 11 cases (12%) were diagnosed as HER2-positive IBrC. This is in line with epidemiological data, which indicates that the HER2-positive phenotype develops in about 15–20% of breast cancer subjects [14]. All HER2-positive patients required the administration of an adjuvant treatment, combining trastuzumab with chemotherapy. Left-sided breast cancers, which are associated with a significant amplification of HER2, were treated significantly more frequently with the trastuzumab antibody (nine cases) than right-sided IBrC (two cases; *p* = 0.0452). Other treatment schemes did not differ with respect to tumor localization. Table 2 summarizes the different therapy patterns used in left- and right-sided cancer patients. 

### 3.3. Treatment Profile in Luminal A IBrC Patients

In order to find potential associations of the laterality with treatment variants, we split the study group into a more homogeneous subgroup by selecting only patients with luminal A IBrC (*n* = 61). In this group, chemotherapy was administered to 14 patients (23%), where 12 women had right-sided breast cancer while two had cancer in the left breast (LRR = 0.16); this difference was significant (*p* = 0.0288). Other treatment procedures failed to show statistical significance with respect to IBrC laterality, although there was a trend (*p* = 0.0957) in women receiving adjuvant endocrine therapy, with 33 patients of right-sided IBrC and 24 subjects of left-sided cancer (LRR = 0.72), which was undoubtedly associated with a higher incidence of luminal A breast cancer in patients with right-sided breast cancer (Table 3). 

### 3.4. Angiogenic and Hemostatic Profiles of IBrC Patients

Differences in the angiogenic profiles and hemostatic parameters between left- and right-sided breast cancers were also studied. Table 4 displays these data. A significantly higher concentration of VEGF-A was noted in patients with breast cancer localized in the left breast (*p* = 0.0136). Furthermore, lower anti-angiogenic potential expressed by sVEGFR1/VEGF-A and sVEGFR2/VEGF-A ratios in patients with left-sided breast tumor was recorded (*p* = 0.0208; *p* = 0.0068, respectively). Our study demonstrates one more important observation that the soluble forms of the VEGF type 1 and 2 receptors as a single determinant did not differ significantly between the groups. However, juxtaposition of sVEGFR1/VEGF-A and sVEGFR2/VEGF-A ratios revealed statistically significant differences. A significantly higher concentration of PAI-1 was noted in patients with breast cancer localized in the left breast (*p* = 0.0229). No other markers showed significant *p*-values, although there was a tendency toward a higher activity of TFPI (*p* = 0.0985) in women who developed left-breast tumor.

### 3.5. Angiogenic and Hemostatic Profiles of Luminal-A IBrC Patients

The same analysis was performed in the more homogeneous subgroup of luminal-A IBrC patients (Table 5). In this case, patients with left-sided tumors displayed a concentration of VEGF-A which was nearly three-fold higher with respect to their right-sided counterparts (*p* = 0.0005). Juxtaposition of sVEGFR1/VEGF-A and sVEGFR2/VEGF-A ratios revealed significant differences as well (*p* = 0.0104; *p* = 0.0012, respectively).

The activity and concentration of TFPI showed a tendency toward higher levels in patients who developed cancer in the left breast but did not reach significance (*p* = 0.0686; *p* = 0.0722, respectively). This subgroup of luminal A patients, unlike the global group, exhibited no differences in PAI-1 concentrations.

### 3.6. Survival of Patients with IBrC

**Figure 1:**
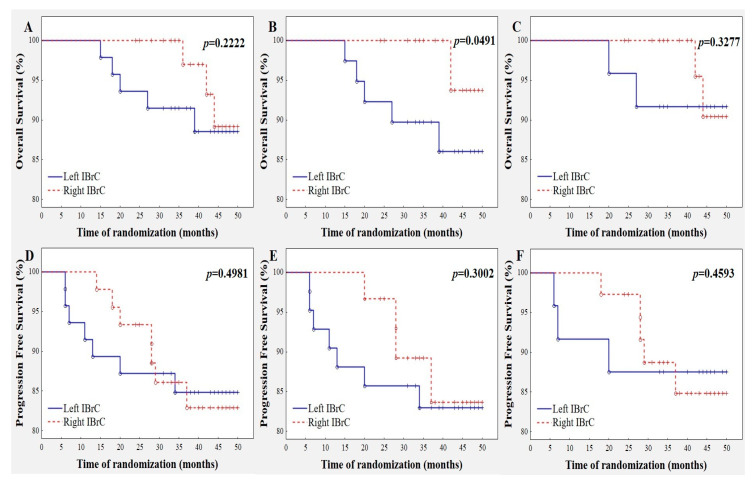
Crude Kaplan–Meier curves for each survival outcome for laterality category. (**A**) Overall survival for the entire study group. (**B**) Breast cancer survival for IBrC cases free of nodal involvement; *p* = 0.0491. (**C**) Overall survival for luminal-A breast cancer subgroup. (**D**) Progression-free survival for the entire study group. (**E**) Progression-free survival for IBrC cases free of nodal involvement. (**F**) Progression-free survival for luminal-A breast cancer subgroup.

To illustrate the relationship between tumor laterality and survival, we plotted crude Kaplan–Meier curves for progression-free and overall survivals. For the progression-free survival analysis, 14 events occurred. The median follow-up was 42 months (IQR: 36–44). Figure 1 displays the survival curves obtained in our study. Overall survival was 89.37% for left-sided tumors and 93.34% for right-sided cancers (Plot A). Recurrence of the disease in the group of patients with right-sided tumors took place in seven out of 45 (15.55%) women, while left-sided IBrC patients suffered a recurrence in seven out of 47 (14.89%) cases (Plot D). Statistical analysis showed that neither overall survival (Plot A) nor progression-free survival (Plot D) differed with regard to tumor laterality. 

Overall survival in the subgroup of luminal-A IBrC cases was 91.67% for left-sided tumors and 94.59% for right-sided cancers (Plot C). Recurrence of the disease in the right-sided luminal A subgroup took place in five out of 37 (13.51%) women while left-sided IBrC patients exhibited a recurrence in three out of 24 (12.50%) cases (Plot F). As with the global group, no differences with regard to overall survival and progression-free survival were found in luminal-A IBrC patients. 

Lymph node status is a fundamental indicator among prognostic markers designed for predicting spread or overall survival for invasive breast cancer. Thus, we performed a similar survival statistical analysis in subjects with lack of metastasis to regional lymph nodes. The group consisted of 72 cases; 42 patients had left-sided breast cancer and 30 had right-sided ones (Table 1.11). Overall survival for left-sided IBrC cases were 88.09% versus 96.67% in their right-sided counterparts (Plot B) (*p* = 0.0491); hence, patients with left-sided tumors presented 8.58% higher risk of disease relapse. However, progression-free survival did not differ between subgroups (Plot E).

### 3.7. Predictive Factors for Left/Right-Sided Invasive Breast Cancer as Assessed by Multivariate Cox’s Proportional Hazards Regression Analysis

A multivariate Cox hazard analysis, which takes into account the function of time, revealed that, in right-sided tumors, women’s BMI (hazard ratio (HR), 0.03; 95% confidence interval (CI), 0.00–0.89; *p* = 0.0316) and tumor diameter (HR, 17.38; 95% CI, 1.88–160.41; *p* = 0.0146), were significant predictive factors for disease relapse in BrC subjects. The analysis showed that only right-sided breast cancer subjects with a lower BMI and larger tumors have a significantly higher risk of recurrent events. Similar dependencies were not detected in left-sided IBrC (Table 6). Thus, the predictive value of clinicopathological characteristics depends on tumor laterality.

### 3.8. Prognostic Values of Angiogenic and Hemostatic Parameters with Respect to Disease Relapse

Linear regression analysis showed that the concentration of VEGF-A was positively associated with recurrence of the disease (*β* = 0.3493, *p* = 0.0166). Additionally, there was a significant linear negative relationship between the concentration of the soluble form of VEGF receptor type 2 (sVEGFR2) and disease recurrence (*β* = −0.3937, *p* = 0.0164). Both relationships were noted in left-sided tumors. Other angiogenic parameters did not show a prognostic value with respect to tumor laterality (Table 7), nor did hemostatic factors (Table 8).

## 4. Discussion and Conclusions

Women’s breasts show bilateral divergent tissue volume, structure, position, arterial and venous supply, and lymphatic drainage, which are associated with breast asymmetry. This laterality is precisely regulated during fetal development and may affect the prevalence of disease [2,3,4,5]. Mammary glands are constantly exposed to estrogen/progesterone fluctuation during the menstrual cycle, environmental factors such as trauma, infection, or surgery, and genetic implications (brain asymmetry and breast cancer susceptibility may both be affected by genes at chromosome locus 11q22–23), which predispose to cancer development [2,4]. Breast cancer is not an exception to these divergences in laterality. To date, no study attempted to analyze differences lateral in breast cancer laterality with regard to angiogenic and hemostatic profiles, as well as prognostic values in IBrC. The present work analyzed these angiogenic and hemostatic profiles in right- and left-sided IBrC in relation with clinical and pathological determinants. This work shows that tumor location in patients with non-metastatic breast cancer plays an important role in overall survival. It would suggest that tumor localization predicts survival.

### 4.1. Clinicopathological Features and Tumor Laterality

We calculated the relative rate of the left- and right-breast involvement in newly diagnosed IBrC patients in order to evaluate whether there was a relevant dominance of cancer on a particular side. Data on this issue are currently sparse. We found a similar breast cancer distribution with a left-to-right ratio of 1.04, which is in line with most previous reports [4,15,16]. However, Fatima et al. noted a significantly higher incidence rate in left-sided breast cancer than in the right-sided ones, and an explanation of this fact is still equivocal [17]. It was suggested that breast cancer incidence laterality is associated with ethnicity or the place of residence. Our study patients, like those of Rutter et al. [16], are Caucasian, but this was not always the case, as similar results were obtained in Egyptian women [15]. 

In our study, we observed that, of the 20 cases with confirmed nodal metastases, 75% suffered right-sided breast cancer and 25% had left-sided breast cancer, which is in line with Nouh et al.’s and Dane et al.’s studies [18,19]. These authors showed that metastases in regional lymph nodes were observed less commonly in left-sided breast IBrC cases than in right-sided cancer. The authors claimed that this was related to a higher activity of the right hand [18]. It follows from this that right-sided breast cancers are more prone to having higher nodal involvement, which is a well-known indicator for recurrence of the disease [19]. Our study provides further information by showing that right-sided breast cancer patients tend to develop primary tumors which are bigger in size. Importantly, in right-sided tumors, the tumor diameter is a very strong predictor of disease relapse. It is worth emphasizing that patients with right-sided luminal A tumors were more aggressively treated in term of surgery, chemo-, radio-, and brachytherapy, and hormone therapy than left located cancers. Altogether, these data suggest that the laterality of breast cancer is related to the metastatic character of the tumor. Analyses carried out so far demonstrate that the strongest predictors of the disease relapse are nodal involvement and tumor size. Fatima et al. also suggested that the prognosis is associated with the fact that patients with right-sided breast cancer are younger than those with left-sided breast cancer [17]. It is well known that cancers developed at younger ages are more aggressive. Perhaps an additional negative factor, which influences progression-free survival, was the treatment approach. 

We found significantly higher occurrence of HER2-positive scores in left-sided IBrC than in right breasts. Tumors with overexpression of HER2 are indicative of a more invasive character including intensive proliferation and angiogenesis, resistance to apoptosis, and a less effective response to chemotherapy. Furthermore, application of HER2-targeted therapy improves treatment efficacy and outcome in HER2-positive breast cancer women [14,20]. Our findings suggest that right- and left-sided tumors present divergent tendency in relation to molecular subtypes of IBrC, since right-sided tumors demonstrate a tendency to be the luminal A (40% vs. 26%) subtype of IBrC, while left-sided tumors had more luminal B, HER2-positive, and triple-negative (25% vs. 9%) cases. Additionally, lower and higher advancement (G1 and G3) of tumors was observed in left-sided IBrC than in right-sided tumors. Interestingly, according to Cox’s analysis, we showed that only in right-sided IBrC subjects do lower BMI and larger tumors demonstrate prognostic values. Apparently, distinct prognostic factors are involved in IBrC laterality.

Bilateral symmetry of paired organs (i.e., kidneys, ovaries, testicles, or breasts) is well documented. Breast development is associated with essential estrogen impact. Furthermore, breast density depends on estrogen concentration under a menstrual cycle. Some women are more prone to “fluctuating asymmetry” (FA) and, in consequence, one breast is more predisposed to cancer development. FA is more noticeable in larger breasts than smaller ones, as well as in nulliparous women [7,21] It is well-established that estrogens present strong mitogenic properties, as they stimulate endothelial cell activation, migration, proliferation, and growth, which can subsequently lead to acceleration of neoangiogenesis [1,7]. Therefore, a lower number of menses, due to pregnancy and lactation, is confirmed as a protective barrier against breast cancer development [7]. 

### 4.2. Angiogenic Parameters and Tumor Laterality

The main finding of this study is the significantly higher concentration of VEGF-A and the lower anti-angiogenic potential as expressed by sVEGFR1/VEGF-A and sVEGFR2/VEGF-A ratios in patients who developed IBrC in the left breast. Importantly, these angiogenic parameters demonstrated prognostic values in this condition. Indeed, a high VEGF-A concentration is significantly associated with poor survival in breast cancer patients [22,23], but the concentration of the soluble form of VEGF receptor type 2 negatively influenced disease relapse [23]. Those observations are consistent with our findings based on a linear regression model. The rationale of using sVEGFR1/VEGF-A and sVEGFR2/VEGF-A ratios as indicators of anti-angiogenic status is based on the assumption that sVEGFR1 and sVEGFR2 are major suppressors of circulating VEGF bioavailability [23,24,25]. Interestingly, our study shows that the soluble forms of the VEGF type 1 and 2 receptors as single determinants did not differ significantly between the groups. However, juxtaposition of sVEGFR1/VEGF-A and sVEGFR2/VEGF-A ratios revealed statistically significant differences. Further confirmation of the mentioned divergences was obtained when the same comparative study was performed in a more homogeneous group of patients, i.e., the luminal A subtype of breast cancer cases. Thus, our study suggests that the angiogenic potential of breast cancer is related to the “fluctuating asymmetry” and varies depending on breast cancer localization (left or right breast). Patients with tumors localized in the left breast present essential activation of angiogenic process and exhibit lower anti-angiogenic capacity. VEGF overexpression is associated with microvessel density, metastasis, tumor growth, poor prognosis [26], and worse patient outcome [27]. Golding et al. noted that heparin-binding epidermal growth factor-like growth factor (HB-EGF) is more intensively expressed in murine embryonic myotomes on the left side than the right side. HB-EGF is a powerful mitogen and chemoattractant for keratinocytes, fibrocytes, and smooth muscle cells. HB-EGF is highly expressed in breast cancer [28]. Wilting and Hagedorn suggested that an uneven left–right activity of HB-EGF and its receptors impact laterality in breast cancer development [3]. Thus, vascular endothelial growth factor is a major agent in angiogenesis regulation in malignant processes. A higher level of VEGF-A, together with the lower anti-angiogenic potential of left-sided IBrC women, suggests the existence of increased vessel formation within the tumor and more nutrition of cancer cells with poorer future prognosis. 

### 4.3. Hemostatic Factors with Respect to Tumor Laterality

In addition to angiogenesis, the hemostasis system is importantly involved in tumor growth. In our study, we observed a higher concentration of plasminogen activator inhibitor type 1 (PAI-1) and a tendency toward a higher activity of tissue factor pathway inhibitor (TFPI) in left-sided IBrC. PAI-1 suppresses fibrinolysis and enhances thrombotic microenvironment [29,30]. On the other hand, TFPI is crucial for thrombosis prevention [31]. Higher TFPI activity may have generated a higher inhibitory potential on the initial phase of the extrinsic coagulation pathway, as suggested by the lower tissue factor activity in patients with a left-breast tumor. This was further confirmed by the higher concentration of TFPI in luminal A left-breast cancer patients. Interestingly, in the luminal-A IBrC subgroup of patients, the significant differences in PAI-1 concentration between left- and right-sided cancer women were lost. Furthermore, both agents present an inverse impact on tumor development. High expression of TFPI is predictive of increased overall survival in patients with breast cancer. Tinholt et al. suggested the potential impact of TFPI as a breast cancer development inhibitor [31]. Moreover, Xu et al. observed that patients with higher expression of TFPI showed longer disease-free survival (DFS). The authors claimed that subjects with lower TFPI expression are more prone to risk of disease recurrence [32]. Tinholt et al. noted that the levels of TFPI exhibit an inclination to be lower in larger tumors, grade-3 tumors, and triple-negative tumors. These results indicate that TFPI is inversely associated with breast cancer invasion and metastasis. Since TFPI suppresses vascular endothelial growth factor (VEGF), plasminogenesis, and metalloprotease, it controls tumor growth and metastasis [31]. Unfortunately, tumor spread, resistance to apoptosis by cancer cells, and proangiogenic activity are associated with the upregulation of PAI-1 [29,33]. Moreover, from the clinical point of view, elevated levels of PAI-1 are related to increased tumor stage, as well as worse relapse-free survival and poor overall survival in breast cancer cases [33,34]. Ferroni et al. suggested that higher PAI-1 concentration might be a negative prognostic marker of breast cancer progression. Perhaps the lack of significant difference in regard to PAI-1 concentration in the homogeneous group of patients with luminal A IBrC obtained in our study predicts a better future outcome in these subjects [34]. 

### 4.4. Survival Analysis with Respect to Tumor Laterality

We found that, in the IBrC group with a lack of metastases to regional lymph nodes, the recurrence rates for left-sided cases were significantly higher versus their right-sided counterparts. Thus, patients with left-sided breast tumors present a higher risk of disease recurrence compared to right-sided IBrCs. Our findings are consistent with Darby et al., which demonstrated that left-sided tumors are associated with worse normal tissue toxicities and survival [35]. Interestingly, Amer did not observe a statistically significant difference in survival between patients with left or right breast cancers, as well as in those with or without a family history of cancer [4]. It is well known that the main cause of death in breast cancer is not the primary tumor, but the incident of lethal metastases to vital organs. Proper diagnosis and prediction of invasive breast cancer using simple clinical features and easily accessible laboratory data will help in improving the patient’s prognosis. Thus, our results on survival and laterality further support the idea of considering tumor laterality as a prognostic feature.

Our study has several strengths, including its prospective nature and the advantage of working on a well-characterized IBrC population with respect to clinicopathological features and treatment patterns. Additionally, none of the enrolled patients were lost to follow-up, and everyone received the scheduled treatment. The data related to cause of death were provided; thus, a cancer-specific survival analysis was possible to conduct. The findings must be seen in the light of some limitations. The study was performed in patients from a single center due to resource limitations. There was a lack of information regarding *BRCA1* and *BRCA2* gene mutations. A weakness of the study was its relatively small sample size, especially for some subgroups, and this could have affected our ability to highlight some differences. Nevertheless, it should be highlighted that selection of IBrC at the early stage (I–IIB) was quite challenging. Another limiting factor was the relatively short follow-up time (the median follow-up was 42 months). Nonetheless, our results reveal potentially valuable information that breast tumor laterality may serve as an indicator of future prognosis.

In summary, our study confirms the heterogeneous character of breast cancer. Localization of breast cancer can be split in IBrC patients with better or worse prognosis. Thus, left-sided breast cancer patients presented 8.58% higher risk of disease relapse provided no lymph node metastases developed. Additionally, our study revealed a specific clinicopathological pattern for left- and right-sided IBrC, since only right-sided breast cancer subjects with a lower BMI and larger tumors demonstrated a significantly higher risk of recurrent events. Furthermore, angiogenic and hemostatic profiles can describe the differential behavior of carcinomas with regard to breast laterality. The concentrations of VEGF-A and sVEGFR2 may serve as easy applicable biomarkers with respect to disease recurrence, but only in left-sided tumors. We suggest including tumor location in the diagnostic routine and consider laterality as a prognostic factor of IBrC. Nevertheless, further studies are required to prospectively evaluate the clinical value of breast cancer laterality. In fact, we propose that the use of similar approaches to those described in the current study may be useful for better understanding the potential link between heterogeneity in breast laterality with the malignant behavior and prognosis of breast cancer. 

## Figures and Tables

**Table 1:** Clinical presentation of patients according to breast cancer laterality.

	Feature	Left IBrC *n* (%)	Right IBrC *n* (%)	*p*-Values	LRR
1	Total breast cancer patients	47 (51%)	45 (49%)	0.8542	1.04
2	Age < 55 years	23 (25%)	22 (24%)	0.3516	1.04
	Age ≥ 55 years	24 (26%)	23 (25%)		1.04
3	Premenopausal	16 (17%)	14 (15%)	0.7643	1.14
	Postmenopausal	31 (34%)	31 (34%)		1.0
4	Normal weight	20 (22%)	25 (27.2%)	0.1511	0.8
	Overweight	16 (17.4%)	16 (17.4%)		1
	Obese	11 (12%)	4 (4%)		2.75
5	Smokers	13 (14%)	7 (8%)	0.1946	1.85
	Non-smokers	35 (38%)	37 (40%)		0.94
6	Tumor size			0.7163	
	T1	34 (37%)	31 (34%)		1.1
	T2	13 (14%)	14 (15%)		0.93
7	Histology			**0.4794**	
	Ductal (IDC)	43 (47%)	39 (42%)		1.1
	Lobular (ILC)	4 (4%)	6 (7%)		0.66
8	Histological grade			0.0698	
	Grade low (G1)	5 (5%)	1 (1%)		5.0
	Grade moderate (G2)	30 (33%)	37 (40%)		0.81
	Grade high (G3)	12 (13%)	7 (8%)		1.71
9	HER2 (null)	20 (22%)	22 (24%)	**0.0357**	0.9
	HER2 (+)	18 (19%)	20 (22%)		0.9
	HER2 (++)	0 (0%)	1 (1%)		-
	HER2 (+++)	9 (10%)	2 (2%)		4.5
10	Molecular subtypes			**0.0016**	
	Luminal A	24 (26%)	37 (40%)		0.65
	Other molecular subtypes	23 (25%)	8 (9%)		2.87
11	Axillar Lymph Node Status			**0.0083**	
	Negative	42 (46%)	30 (33%)		1.4
	Positive	5 (5%)	15 (16%)		0.33

IBrC—invasive breast cancer, LRR—left-to-right ratio, IDC—invasive ductal carcinoma, ILC—invasive lobular carcinoma, HER2—human epidermal growth factor receptor 2, HER2 (null–2+)—non overexpressed, HER2 (+++)—overexpression; other molecular subtypes included luminal B HER2^−/+^, basal-like, and non-luminal HER2^+^; significant differences are denoted by bold *p*-values, while underlined *p*-values represent closeness to statistical significance.

**Table 2:** Laterality of breast cancer patients and their treatment profiles.

Characteristic	Total IBrC		Left IBrCs		Right IBrCs		*p*-Values	LRR
	n	%	n	%	n	%		
Surgery								
BCS	67	73	37	79	30	67	0.1938	1.23
MRM	25	27	10	21	15	33		0.66
Radiotherapy								
No	17	18	7	15	10	22	0.3653	0.7
Yes	75	82	40	85	35	78		1.14
Brachytherapy								
No	49	53	26	55	23	51	0.6859	1.13
Yes	43	47	21	45	22	49		0.95
Chemotherapy								
No	51	55	24	51	27	60	0.3887	0.88
Yes	41	45	23	49	18	40		1.27
Immunotherapy								
No	81	88	38	41	43	47	**0.0452**	0.88
Yes	11	12	9	10	2	2		4.5
Hormonal therapy								
No	18	20	9	19	9	20	0.9181	1
Yes	74	80	38	81	36	80		1.05

IBrC—invasive breast cancer, LRR—left-to-right ratio, BCS—breast-conserving surgery, MRM—modified radical mastectomy; a significant difference is denoted by bold *p*-values.

**Table 3:** Laterality of luminal A breast cancer patients and their treatment profiles.

Characteristic	Total IBrC		Left IBrC		Right IBrC		*p*-Values	LRR
	n	%	n	%	n	%		
Surgery								
BCS	43	70	18	75	25	68	0.5341	0.72
MRM	18	30	6	25	12	32		0.5
Radiotherapy								
No	11	18	3	12.50	8	22	0.3653	0.37
Yes	50	82	21	87.50	29	78		0.72
Brachytherapy								
No	32	52	14	58	18	49	0.4594	0.77
Yes	29	48	10	42	19	51		0.52
Chemotherapy								
No	47	77	22	92	25	68	**0.0288**	0.88
Yes	14	23	2	8	12	32		0.16
Hormonal therapy								
No	4	7	0	0	4	11	0.0957	0
Yes	57	93	24	100	33	89		0.72

IBrC—invasive breast carcinoma, LRR—left-to-right ratio, BCS—breast-conserving surgery, MRM—modified radical mastectomy; a significant difference is denoted by bold *p*-values; underlined *p*-values represent closeness to statistical significance.

**Table 4:** Angiogenic, hemostatic, and inflammatory biomarkers according to breast cancer laterality in all breast cancer patients.

Parameter (units)	Left IBrC	Right IBrC	*p*-Values
VEGF-A concentration (pg/mL)	87.81 48.91–144.46	51.56 32.47–93.60	**0.0136**
SDF-1α concentration (ng/mL)	0.42 0.39–0.50	0.42 0.40–0.49	0.4617
sVEGFR1 concentration (pg/mL)	24.45 19.20–70.37	30.99 18.09–79.80	0.3326
sVEGFR2 concentration (pg/mL)	9778.25 7681.51–11,588.35	9369.43 8181.11–12,483.70	0.5509
Heparanase concentration (pg/mL)	185.98 137.80–276.15	165.88 135.68–252.87	0.4274
TF concentration (pg/mL)	563.96 400.94–718.70	521.76 400.86–710.40	0.6425
TF activity (pM)	13.60 9.81–29.59	14.10 12.31–25.20	0.4029
TFPI concentration (ng/mL)	44.80 39.08–61.64	43.00 33.40–55.00	0.1849
TFPI activity (UI/mL)	1.38 1.14–1.58	1.20 1.10–1.46	0.0985
vWF concentration (mU/mL)	600.00 382.10–811.80	569.90 470.00–737.70	0.8663
t-PA concentration (ng/mL)	5.41 3.86–7.16	5.26 3.86–6.34	0.6553
PAI-1 concentration (ng/mL)	43.76 33.96–54.56	36.20 24.40–42.91	**0.0229**
YKL-40 concentration (ng/mL)	2.03 1.44–2.85	1.89 1.42–2.57	0.6099
sVEGFR1/ VEGF-A ratio	0.34 0.12–1.01	0.63 0.37–1.95	**0.0208**
sVEGFR2/ VEGF-A ratio	126.66 72.59–214.08	209.34 116.05–286.84	**0.0068**
TF/TFPI activity ratio	8.98 5.78–30.40	12.16 9.73–24.13	0.2919
TF/TFPI concentration ratio	11.19 8.43–17.30	11.75 9.37–17.58	0.5414
PAI-1/t-PA concentration ratio	0.13 0.08–0.16	0.14 0.07–0.26	0.2563

IBrC—invasive breast cancer, VEGF-A—vascular endothelial growth factor A, SDF-1α—stromal cell-derived factor-1α, sVEGFR1—soluble form of vascular endothelial growth factor receptor type 1, sVEGFR2—soluble form of vascular endothelial growth factor receptor type 2, TF—tissue factor, TFPI—tissue factor pathway inhibitor, vWF—von Willebrand factor, t-PA—tissue plasminogen activator, PAI-1—plasminogen activator inhibitor type 1; significant differences are denoted by bold *p*-values; underlined *p*-values represent closeness to statistical significance.

**Table 5:** Angiogenic, hemostatic, and inflammatory biomarkers according to breast cancer laterality in luminal A breast cancer patients.

Parameter (units)	Left IBrC	Right IBrC	*p*-Values
VEGF-A concentration (pg/mL)	117.85 61.91–179.38	44.51 30.70–76.83	**0.0005**
SDF-1α concentration (ng/mL)	0.43 0.39–0.53	0.42 0.40–0.52	0.9385
sVEGFR1 concentration (pg/mL)	22.34 17.34–73.91	32.09 24.24–84.48	0.1236
sVEGFR2 concentration (pg/mL)	10,492.32 7961.91–12,422.06	9088.04 7946.69–11,955.80	0.5498
Heparanase concentration (pg/mL)	183.71 137.80–286.91	165.88 131.46–252.87	0.4943
TF concentration (pg/mL)	537.87 422.63–640.08	539.93 400.86–726.12	0.5628
TF activity (pM)	11.32 8.95–27.16	14.06 12.31–25.19	0.1448
TFPI concentration (ng/mL)	50.62 43.52–62.28	44.04 34.34–55.68	0.0722
TFPI activity (UI/mL)	1.37 1.25–1.60	1.18 1.10–1.40	0.0686
vWF concentration (mU/mL)	569.90 382.10–811.80	569.90 460.00–737.70	0.7797
t-PA concentration (ng/mL)	4.51 3.45–6.91	5.11 3.44–7.12	0.8592
PAI-1 concentration (ng/mL)	41.14 33.83–50.96	35.12 22.66–44.60	0.1325
YKl-40 concentration (ng/mL)	2.02 1.38–3.21	1.89 1.42–2.57	0.7812
sVEGFR1/ VEGF-A ratio	0.20 0.08–0.70	0.69 0.45–1.01	**0.0104**
sVEGFR2/ VEGF-A ratio	88.47 62.42–179.83	219.31 139.35–295.71	**0.0012**
TF/TFPI activity ratio	7.43 5.75–27.86	11.54 9.73–24.13	0.1535
TF/TFPI concentration ratio	10.29 7.59–14.10	11.75 9.36–18.46	0.1448
PAI-1/t-PA concentration ratio	0.13 0.08–0.16	0.16 0.07–0.26	0.2941

IBrC—invasive breast cancer, VEGF-A—vascular endothelial growth factor A, SDF-1α—stromal cell-derived factor-1α, sVEGFR1—soluble form of vascular endothelial growth factor receptor type 1, sVEGFR2—soluble form of vascular endothelial growth factor receptor type 2, TF—tissue factor, TFPI—tissue factor pathway inhibitor, VWF—von Willebrand factor, t-PA—tissue plasminogen activator, PAI-1—plasminogen activator inhibitor type 1; significant differences are denoted by bold *p*-values; underlined *p*-values represent closeness to statistical significance.

**Table 6:** Multivariate Cox’s hazard ratios for progression-free survival according to selected clinicopathological features with respect to left- and right-sided tumors.

Marker	Left IBrC		Right IBrC	
	HR (95% CI)	*p-Values*	HR (95% CI)	*p-Values*
Molecular subtypes (Luminal A vs. other molecular subtypes)	0.72 (0.13/3.82)	0.6963	0.08 (0.01/1.15)	0.1002
BMI (≤25 kg/m^2^ vs. >25 kg/m^2^)	0.40 (0.09/1.86)	0.2424	0.03 (0.00/0.89)	**0.0316**
Age (<55 years vs. ≥55 years)	1.46 (0.28/7.67)	0.6560	0.32 (0.07/1.54)	0.3120
Tumor diameter (T1 vs. T2)	4.36 (0.93/20.40)	0.0618	17.38 (1.88/160.41)	**0.0146**

IBrC—invasive breast cancer, HR—hazard ratio, BMI—body mass index, T1—<2 cm, T2—≥ 2 cm < 5 cm; other molecular subtypes include luminal B HER2^−/+^, basal-like, and non-luminal HER2^+^; significant values are denoted by bold *p*-values; underlined *p*-values represent closeness to statistical significance.

**Table 7:** Prognostic value of angiogenic parameters with respect to tumor laterality assessed by linear regression.

Parameter		Left IBrC			Right IBrC	
	Unstandardized B (95% CI)	*β*	*p-Values*	Unstandardized B (95% CI)	*β*	*p-Values*
VEGF-A concentration (pg/mL)	0.0011 (0.0002/0.0019)	0.3493	**0.0166**	0.01 (−0.03/0.03)	0.0308	0.8655
sVEGFR1 concentration (pg/mL)	−0.0018 (−0.0046/0.0010)	−0.2068	0.2055	−0.03 (−0.06/0.01)	−0.2686	0.1716
sVEGFR2 concentration (pg/mL)	−0.0001 (−0.0001/−0.0000)	−0.3937	**0.0164**	−0.02 (−0.07/0.03)	−0.1510	0.4751
Heparanase concentration (pg/mL)	−0.0002 (−0.0006/0.0001)	−0.2246	0.1242	0.01 (−0.03/0.05)	0.1031	0.5832
SDF-1α concentration (ng/mL)	−0.0058 (−0.0173/0.0058)	−0.1424	0.3219	−0.09 (−0.51/0.33)	−0.0759	0.6689
	***R*^2^ = 0.2809**		**0.0233**	***R*^2^ = 0.0820**		**0.7213**

IBrC—invasive breast cancer, VEGF-A—vascular endothelial growth factor A, sVEGFR1—soluble form of vascular endothelial growth factor receptor type 1, sVEGFR2—soluble form of vascular endothelial growth factor receptor type 2, SDF-1α—stromal cell-derived factor-1α; significant differences are denoted by bold *p*-values.

**Table 8:** Prognostic value of hemostatic variables with respect to tumor laterality assessed by linear regression.

Parameter	Left IBrC			Right IBrC		
	Unstandardized B (95% CI)	*β*	*p-Values*	Unstandardized B (95% CI)	*β*	*p-Values*
TF activity (pM)	0.01 (−0.01/0.02)	0.3568	0.0787	0.09 (−0.03/0.22)	0.2524	0.1394
TF concentration (pg/mL)	0.03 (−0.02/0.09)	0.2128	0.2173	−0.02 (−0.09/0.05)	−0.1106	0.5142
TFPI activity (UI/mL)	0.01 (−0.37/0.40)	0.0136	0.9403	−0.01 (−0.35/0.34)	−0.0044	0.9793
TFPI concentration (ng/mL)	0.00 (−0.01/0.01)	0.0918	0.5847	−0.05 (−0.14/0.04)	−0.1978	0.2584
t-PA concentration (ng/mL)	−0.02 (−0.07/0.03)	−0.1235	0.4597	0.01 (−0.04/0.06)	0.0614	0.7367
PAI-1 concentration (ng/mL)	0.00 (−0.01/0.01)	−0.2615	0.1993	−0.04 (−0.41/0.33)	−0.0391	0.8186
vWF concentration (mU/mL)	−0.01 (−0.06/0.03)	−0.0952	0.5639	−0.03 (−0.10/0.03)	−0.1878	0.2672
	*R*^2^ = 0.1636		0.4824	*R*^2^ = 0.1490		0.5735

IBrC—invasive breast cancer, TF—tissue factor, TFPI—tissue factor pathway inhibitor, t-PA—tissue plasminogen activator, PAI-1—plasminogen activator inhibitor type 1, vWF—von Willebrand factor; underlined *p*-values represent closeness to statistical significance.

## References

[1] Von Fellenberg R. (1940). Schweizerische Erhebung uber maligne Tumoren 1933–1935 [Switzerland. Survey of malignant tumors]. Bull. des Eidgenoss. Gesundheitsamt.

[2] Chen J.H., Chan S., Yeh D.C., Fwu P.T., Lin M., Su M.Y. (2013). Response of bilateral breasts to the endogenous hormonal fluctuation in a menstrual cycle evaluated using 3D MRI. Magn. Reson. Imaging.

[3] Wilting J., Hagedorn M. (2011). Left-right asymmetry in embryonic development and breast cancer: Common molecular determinants?. Curr. Med. Chem..

[4] Amer M.H. (2014). Genetic factors and breast cancer laterality. Cancer Manag. Res..

[5] Roychoudhuri R., Putcham V., Møller H. (2006). Cancer and laterality: A study of the five major paired organs (UK). Cancer Causes Control..

[6] Erendeeva L.E., Zav’yalova M.B., Slonimskaya E.M., Perelmuter V.M. (2002). Influence of functional asymmetry on the forecast of a breast cancer. Byul Sib. Med..

[7] Chan S., Su M.Y., Lei F.J., Wu J.P., Lin M., Nalcioglu O., Feig S.A., Chen J.H. (2011). Menstrual cycle-related fluctuations in breast density measured by using three-dimensional MR imaging. Radiology.

[8] Bao J., Yu K.D., Jiang Y.Z., Shao Z.M., Di G.H. (2014). The effect of laterality and primary tumor site on cancer-specific mortality in breast cancer: A SEER population-based study. PLoS ONE.

[9] Eisenreich A., Bolbrinker J., Leppert U. (2016). Tissue Factor: A conventional or alternative target in cancer therapy. Clin. Chem..

[10] Hu Z., Cheng J., Xu J., Ruf W., Lockwood C.J. (2017). Tissue factor is an angiogenic-specific receptor for factor VII-targeted immunotherapy and photodynamic therapy. Angiogenesis.

[11] Wang Y., Sang A., Zhu M., Zhang G., Guan H., Ji M., Chen I. (2016). Tissue factor induces VEGF expression via activation of the Wnt/β-catenin signaling pathway in ARPE-19 cells. Mol. Vis..

[12] Rhone P., Bielawski K., Ziołkowska K., Rość D., Ruszkowska-Ciastek B. (2019). Low pre-treatment count of circulating endothelial progenitors as a prognostic biomarker of the high risk of breast cancer recurrence. J. Clin. Med..

[13] Rhone P., Ruszkowska-Ciastek B., Bielawski K., Brkic A., Zarychta E., Góralczyk B., Roszkowski K., Rość D. (2018). Comprehensive analysis of haemostatic profile depending on clinicopathological determinants in breast cancer patients. Biosci. Rep..

[14] Elster N., Toomey S., Fan Y., Cremona M., Morgan C., Weiner Gorzel K., Bhreathnach U., Milewska M., Murphy M., Madden S. (2018). Frequency, impact and a preclinical study of novel ERBB gene family mutations in HER2-positive breast cancer. Ther. Adv. Med. Oncol..

[15] Zeeneldin A.A., Ramadan M., Elmashad N., Fakhr I., Diaa A., Mosaad E. (2013). Breast cancer laterality among Egyptian patients and its association with treatments and survival. J. Egypt. Natl. Canc. Inst..

[16] Rutter C.E., Chagpar A.B., Evans S.B. (2014). Breast cancer laterality does not influence survival in a large modern cohort: Implications for radiation-related cardiac mortality. Int. J. Radiat. Oncol. Biol. Phys..

[17] Fatima N., Zaman M.U., Maqbool A., Khan S.H., Riaz N. (2013). Lower incidence but more aggressive behavior of right sided breast cancer in Pakistani women: Does right deserve more respect?. Asian Pac. J. Cancer Prev..

[18] Nouh M.A., Ismail H., El-Din N.H., El-Bolkainy M.N. (2004). Lymphnode metastasis in breast carcinoma: Clinicopathologic correlations in 3747 patients. J. Egypt. Natl. Canc. Inst..

[19] Dane S., Yildirim S., Koc M., Aktan M., Gundogdu C. (2008). Asymmetries in breast cancer lateralization and both axillary lymph node number and metastatic involvement. Lymphology.

[20] Roth J., Bajaj P., Sullivan S.D., Reyes C., Antao V., Stein A., Mahtani R., Ramsey S. Survival gains from advances in first-line systemic therapy for HER2 overexpressing metastatic breast cancer in the U.S., 1995–2015. Abstract 263P. Proceedings of the ESMO Congress 2017.

[21] Scutt D., Lancaster G.A., Manning J.T. (2006). Breast asymmetry and predisposition to breast cancer. Breast Cancer Res..

[22] Linderholm B.K., Hellborg H., Johansson U., Elmberger G., Skoog L., Lehtiö J., Lewensohn R. (2009). Significantly higher levels of vascular endothelial growth factor (VEGF) and shorter survival times for patients with primary operable triple-negative breast cancer. Ann. Oncol..

[23] Wu F.T., Stefanini M.O., Gabhann F.M., Kontos C.D., Annex B.H., Popel A.S. (2010). A systems biology perspective on sVEGFR1: Its biological function, pathogenic role and therapeutic use. J. Cell. Mol. Med..

[24] Aweimer A., Stachon T., Tannapfel A., Köller M., Truss M.C., Stachon A. (2012). Regulation of soluble VEGFR-2 secreted by microvascular endothelial cells derived from human BPH. Prostate Cancer Prostatic Dis..

[25] Ebos J.M., Lee C.R., Bogdanovic E., Alami J., Van Slyke P., Francia G., Xu P., Mutsaers A.J., Dumont D.J., Kerbel R.S. (2008). Vascular endothelial growth factor–mediated decrease in plasma soluble vascular endothelial growth factor receptor-2 levels as a surrogate biomarker for tumor growth. Cancer Res..

[26] Aoyagi Y., Iinuma H., Horiuchi A., Shimada R., Watanabe T. (2010). Association of plasma VEGF-A, soluble VEGFR-1 and VEGFR-2 levels and clinical response and survival in advanced colorectal cancer patients receiving bevacizumab with modified FOLFOX6. Oncol. Lett..

[27] Martins S.F., Reis R.M., Rodrigues A.M., Baltazar F., Filho A.L. (2011). Role of endoglin and VEGF family expression in colorectal cancer prognosis and anti-angiogenic therapies. World J. Clin. Oncol..

[28] Golding J.P., Tsoni S., Dixon M., Yee K.T., Partridge T.A., Beauchamp J.R., Gassmann M., Zammit P.S. (2004). Heparin-binding EGF-like growth factor shows transient left-right asymmetrical expression in mouse myotome pairs. Gene Expr. Patterns.

[29] Placencio V.R., DeClerck Y.A. (2015). Plasminogen activator inhibitor-1 in cancer: Rationale and insight for future therapeutic testing. Cancer Res..

[30] Mashiko S., Kitatani K., Toyoshima M., Ichimura A., Dan T., Usui T., Ishibashi M., Shigeta S., Nagase S., Miyata T. (2015). Inhibition of plasminogen activator inhibitor-1 is a potential therapeutic strategy in ovarian cancer. Cancer Biol. Ther..

[31] Tinholt M., Vollan H.K.M., Sahlberg K.K., Jernström S., Kaveh F., Lingjærde O.C., Kåresen R., Sauer T., Kristensen V., Børresen-Dale A.L. (2015). Tumor expression, plasma levels and genetic polymorphisms of the coagulation inhibitor TFPI are associated with clinicopathological parameters and survival in breast cancer, in contrast to the coagulation initiator TF. Breast Cancer Res..

[32] Xu C., Wang H., He H., Zheng F., Chen Y., Zhang J., Lin X., Ma D., Zhang H. (2013). Low expression of TFPI-2 associated with poor survival outcome in patients with breast cancer. BMC Cancer.

[33] Lal I., Dittus K., Holmes C.E. (2013). Platelets, coagulation and fibrinolysis in breast cancer progression. Breast Cancer Res..

[34] Ferroni P., Roselli M., Portarena I., Formica V., Riondino S., LA Farina F., Costarelli L., Melino A., Massimiani G., Cavaliere F. (2014). Plasma plasminogen activator inhibitor-1 (PAI-1) levels in breast cancer—relation- ship with clinical outcome. Anticancer Research.

[35] Darby S.C., Cutter D.J., Boerma M., Constine L.S., Fajardom L.F., Kodama K., Mabuchi K., Marks L.B., Mettler F.A., Pierce L.J. (2010). Radiation-related heart disease: Current knowledge and future prospects. Int. J. Radiat. Oncol. Biol. Phys..

